# Overview of human health effects related to glyphosate exposure

**DOI:** 10.3389/ftox.2024.1474792

**Published:** 2024-09-18

**Authors:** Flavia Silvia Galli, Marta Mollari, Valentina Tassinari, Cristian Alimonti, Alessandro Ubaldi, Camilla Cuva, Daniele Marcoccia

**Affiliations:** ^1^ Istituto Zooprofilattico Sperimentale del Lazio e della Toscana “M. Aleandri”, Rome, Italy; ^2^ Department of Experimental Medicine, University of Rome Tor Vergata, Rome, Italy

**Keywords:** glyphosate, plant protection products, mutagenic, cancer, human health and reproduction

## Abstract

Glyphosate is a chemical compound derived from glycine, marketed as a broad-spectrum herbicide, and represents one of the most widely used pesticides in the world. For a long time, it was assumed that glyphosate was harmless, either due to its selective enzymatic acting method on plants, and because commercial formulations were believed to contain only inert chemicals. Glyphosate is widely spread in the environment, the general population is daily exposed to it via different routes, including the consumption of both plant, and non-plant based foods. Glyphosate has been detected in high amounts in workers’ urine, but has been detected likewise in bodily fluids, such as blood and maternal milk, and also in 60%–80% of general population, including children. Considering its massive presence, daily exposure to glyphosate could be considered a health risk for humans. Indeed, in 2015, the IARC (International Agency for Research on Cancer) classified glyphosate and its derivatives in Group 2A, as probable human carcinogens. In 2022, nevertheless, EFSA (European Food Safety Authority) stated that the available data did not provide sufficient evidence to prove the mutagenic/carcinogenic effects of glyphosate. Therefore, the European Commission (EC) decided to renew the approval of glyphosate for another 10 years. The purpose of this review is to examine the scientific literature, focusing on potential risks to human health arising from exposure to glyphosate, its metabolites and its commercial products (e.g., Roundup^®^), with particular regard to its mutagenic and carcinogenic potential and its effects as endocrine disrupter (ED) especially in the human reproductive system.

## 1 Introduction

**FIGURE 1:**
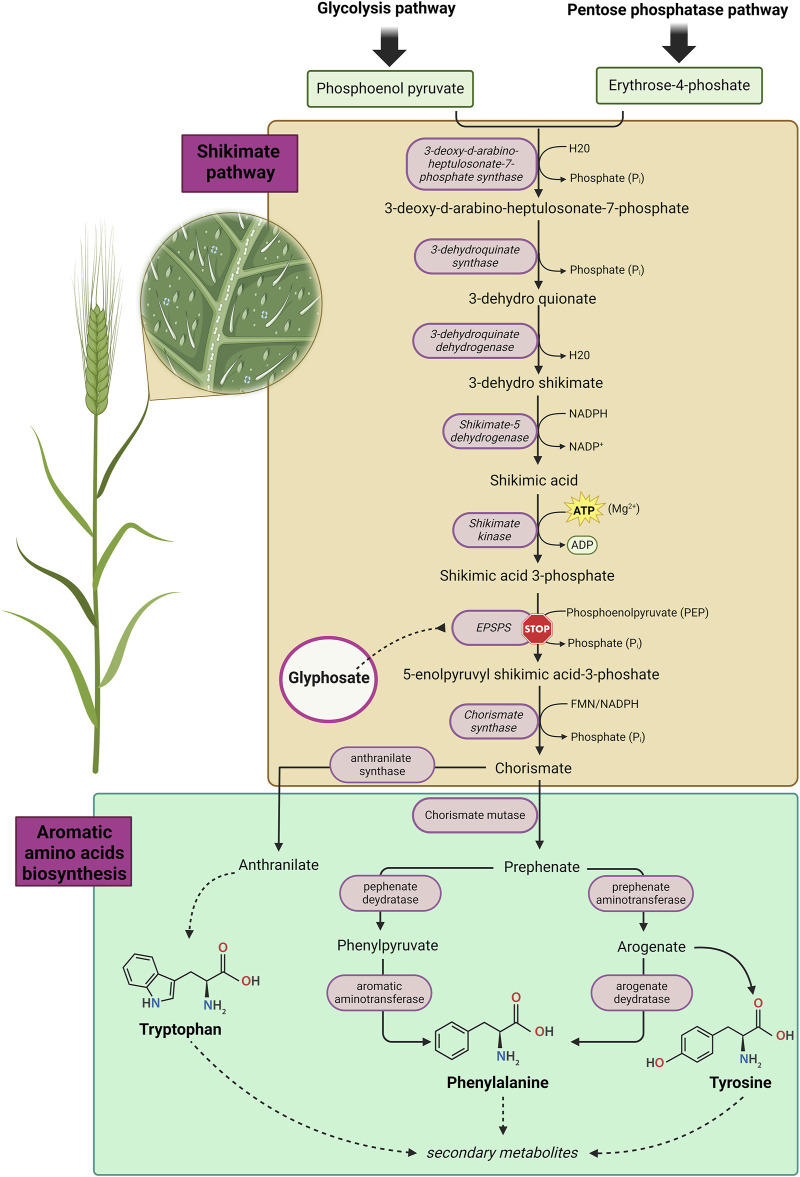
The shikimate pathway in plants. Image has been created with BioRender.com.

Glyphosate (GLY), or N-(phosphonomethyl) glycine, is an organophosphorus compound used as a broad-spectrum non-selective herbicide, considered the most used worldwide (Chaufan et al., 2014; Ferrante et al., 2023). The action of GLY as herbicide is exploited by inhibiting 5-enolpyruvylshikimate-3-phosphate synthase (EPSP), an enzyme generated by plants and microorganisms (Peillex and Pelletier, 2020; Leino et al., 2021). The exertion of this enzyme is achieved by catalysing the condensation reaction between phosphoenolpyruvate and shikimate-3-phosphate (Kanissery et al., 2019; Schwedt et al., 2023). The EPSP inhibition prevents biosynthesis of essential aromatic amino acids (AAAs) such as phenylalanine, tyrosine, and tryptophan), present in chloroplasts and leading to plant deaths (Figure 1). The molecule of GLY was first synthesized in 1950, by the Swiss chemist Henry Martin (Ferrante et al., 2023) and its first authorization dates back to 1974, when Monsanto was granted permission by EPA (Environmental Protection Agency) to market GLY for agricultural use in United State (U.S.), under the trade name Roundup^®^ (Peillex and Pelletier, 2020; Marino et al., 2021). In Europe, the commercialization was only allowed after 2002, following the consent of the European Commission (EC) (Landrigan and Belpoggi, 2018; Marino et al., 2021). The product Roundup^®^ represents the first glyphosate-based herbicide (GBH). The subsequent marketed products such as Glifloglex^®^, Touchdown^®^ and Glyphogan^®^, are formulations in which GLY is present in mixture with different adjuvants, used to increase the plants’ penetration of the herbicide and to enhance its activity (Altmanninger et al., 2023). In most commercial formulations, GLY is found in both acid and salt form (e.g., glyphosate potassium, isopropylamine, and diammonium salt). The additional salts have no impact on glyphosate’s activity as herbicide, but are used to modify some important properties such as its stability in formulations and its handling (Travlos et al., 2017; Altmanninger et al., 2023). Nowadays, GLY represents the active component of several formulation GBHs, employed to control more than 100 species of weeds and 60 species of perennial weed plants in industrial and residential settings (Munoz et al., 2021; Ferrante et al., 2023). From 1996 to 2012, the U.S. GLY market reached a record 12 million tons used for weed treatment and, according to sales forecasts, this record will be surpassed in the coming years (Benbrook, 2016; Ferrante et al., 2023). The implication is that GBH products represent, with their use in about 140 countries, one of the most widely used class of plant protection products (PPPs) in worldwide (Munoz et al., 2021). Due to its widespread use, GLY is detectable in several matrices including air, water, and foodstuffs, and consequently, it can also be found in biological fluids such as urine, blood, and maternal milk (Yoshioka et al., 2011; Mária Mörtl et al., 2013; Zouaoui et al., 2013; Mercurio et al., 2014; Simonetti et al., 2015; Steinborn et al., 2016; Demonte et al., 2018; Marino et al., 2021; Munoz et al., 2021; Cellier et al., 2022; Connolly et al., 2022; Iohanna Filippi et al., 2024). In addition, GLY has also been detected in beer and children’s breakfast cereals, indicating that exposure is not only occupational (Jansons et al., 2018; Marino et al., 2021; Munoz et al., 2021; Martins-Gomes et al., 2022; Abrams et al., 2024). GLY is also widely used for the treatment of genetically modified crops (GMO), such as cereals and soybeans (Bukowska et al., 2022). As a result, these plants have developed a resistance to GLY, being able to convert it into aminomethylphosphonic acid (AMPA), which is significantly more mobile in soil than GLY (Annett et al., 2014; Bukowska et al., 2022) and consequently can also be detected in sediment, surface water and groundwater (Battaglin et al., 2014; Grandcoin et al., 2017). Several toxicity studies have highlighted effects of AMPA on human red blood cells and its potential to produce chromosomal abnormalities in fish (Guilherme et al., 2014; Wozniak et al., 2021). For a long time, it was assumed that GLY was harmless, as its target is the enzyme EPSP, which is not naturally present in mammals. Furthermore, it was assumed that GLY was degraded to CO_2_ and that its formulations contained chemicals erroneously defined as “inert” (Defarge et al., 2018; Munoz et al., 2021; Ferrante et al., 2023). Instead, several epidemiological studies conducted on humans, have revealed a slight increased incidence in the developing of pathologies such as non-Hodgkin’s lymphomas in professionally exposed GLY workers, like farmers (Meloni et al., 2021; Bukowska et al., 2022; Acquavella, 2023; Kim et al., 2023). In addition, several in *in vitro* studies have indicated that GLY may induce genetic damage, increase oxidative stress, interfere with the estrogen pathway, restrict brain functions, and it has been related to several types of cancer as well (Portier et al., 2016; Nagy et al., 2019; Peillex and Pelletier, 2020; Bukowska et al., 2022). As highlighted, exposure to GLY and its metabolite AMPA, which were found in various crops and food (Vandenberg et al., 2017), could pose a potential risk to human health, due also to the prolonged half-life and long-term environmental contamination of both GLY and AMPA (Battaglin et al., 2014). The aim of this review is to provide an overview about the effects of GLY and its metabolites, with particular regard to their mutagenic/carcinogenic potential and their effects as endocrine disruptors (EDs) in the human reproductive system.

## 2 Glyphosate authorization overview

**FIGURE 2:**
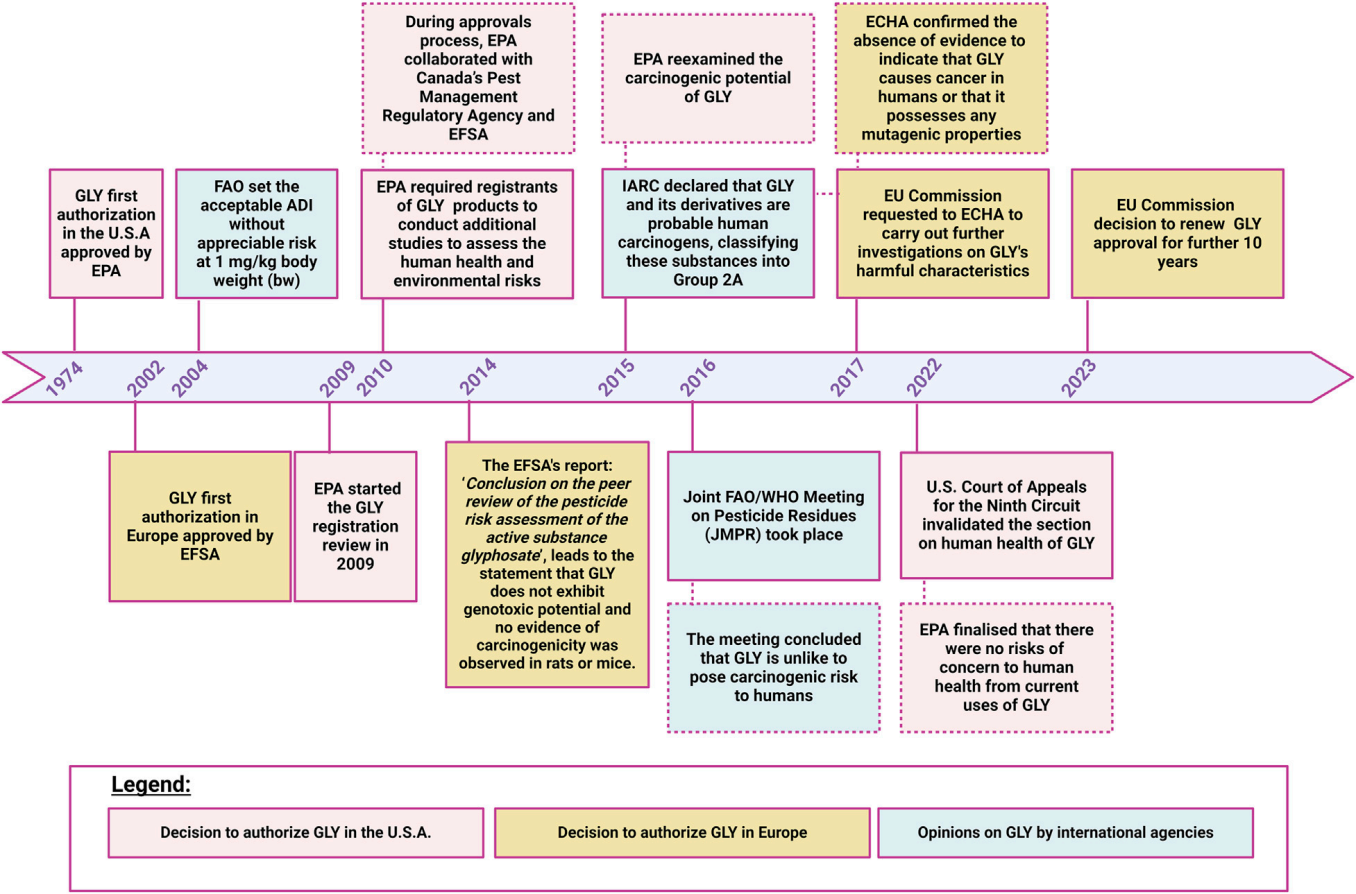
Regulatory timeline of glyphosate. Image has been created with BioRender.com.

GLY was first authorised in 1974 in U.S. following the approval of EPA to place GLY on the market as an herbicide (Peillex and Pelletier, 2020; Marino et al., 2021). After its initial authorization, GLY underwent a renewal cycle in which EPA reviewed and re-evaluated the safety and uses of GLY. During approvals process, EPA cooperated with several worldwide organisations including Pest Management Regulatory Agency (PMRA) and EFSA. Throughout each consultation no concern for human health has been identified by regulatory authorities (Brookes et al., 2017). In 2004, FAO (Food and Agriculture Organisation of the United Nations), due to the presumed low GLY concentration in food, its protracted use over time and its chemical and physical properties, set the GLY acceptable daily intake (ADI) without appreciable risk at 1 mg/kg body weight (bw) (WHO/FAO, 2004). In 2015, IARC declared that GLY and its derivatives are probable human carcinogens, classifying these substances into Group 2A. As a result, EPA re-examined GLY’s potential carcinogenicity and carried out a massive revision of the existing GLY cancer database, including epidemiological, animal carcinogenicity and genotoxicity study data. In 2016, the Joint FAO/WHO Meeting on Pesticide Residues (JMPR) took place. During this meeting, the ADI value for GLY and its metabolites was reconfirmed, and it was also decided that an acute reference dose (ARfD) was unnecessary considering the low toxicity of GLY (WHO/FAO, 2016). The meeting concluded that GLY is unlike to pose carcinogenic risk to humans (Williams et al., 2016). In 2022, the U.S. Court of Appeals for the Ninth Circuit invalidated the section on human health of the GLY ID (mid-term registration review decision) and remanded it to EPA for further analysis and explanation, following which EPA concluded that there were no risks of concern to human health from current uses of GLY. In Europe, the commercialization was only allowed after 2002, following the consent of the European Commission (Landrigan and Belpoggi, 2018; Marino et al., 2021) and EFSA. Since its ‘first approval’, GLY underwent its renewal procedures and is currently authorised in Europe until 2033 (Figure 2). In 2014, the EFSA report “Conclusion on the peer review of the pesticide risk assessment of the active substance glyphosate” concluded that GLY did not present genotoxic potential and no evidence of carcinogenicity was observed in rats or mice. Similarly to what occurred in U.S., after the conclusion of IARC on GLY’s potential carcinogenicity, the EC requested to ECHA to carry out further investigations on GLY’s harmful characteristics prior to any decision regarding its potential renewal at European level. In March 2017, ECHA confirmed the absence of evidence to indicate that GLY causes cancer in humans or that it possesses any mutagenic properties. In May 2022, as in 2017, the ECHA’s Committee for Risk Assessment (RAC) concluded that GLY did not meet the scientific criteria to be classified as a carcinogenic, mutagenic or toxic to reproduction and therefore classifying GLY as a carcinogen is not justified (ECHA, 2017). On September 2023 in a letter addressed to Commissioner - EC, 15 European civil society organizations have expressed their concern regarding the observed tumor incidences in GLY exposed animals and the important mechanistic evidence indicating that GLY induces oxidative stress, highlighting how many studies were not taken into account during the renewal of GLY (EuropeanCommission, 2023). Indeed, even if Regulation 1107/2009 stated that peer-reviewed studies must be collected and evaluated by rapporteur member state (RMS) in the dossier submitted in support of regulatory authorisation or renewal of a pesticide, the EC pesticide regulation does not prescribe that all studies must be considered. As a result, many are omitted and priority is given to those conducted by industries. Moreover, also when published non-industry-sponsored studies are included in the dossier they can be dismissed and excluded from the risk assessment according to Organization for Economic Co-operation and Development (OECD) protocols and Good Laboratory Practice (GLP) rules. On one hand industries studies, due to their compliance with OECD protocols and GLP rule, are deemed determined as sufficiently reliable to be used in the risk assessment (Robinson C and Cavoski, 2020), on the other, according to the EFSA guidance document on implementing Regulation 1107/2009, the fact that a study may not be conducted in accordance with Good Laboratory Practice (GLP) does not imply that the study is irrelevant (EFSA, 2011). Despite the above mentioned inconsistencies, further to consultations involving the EU Commission, ECHA, Member States and EFSA, on 28 November 2023, the European Commission adopted the Implementing Regulation to renew the GLY approval for other 10 years.

## 3 Glyphosate mode of action and its physical and chemical properties

**FIGURE 3:**
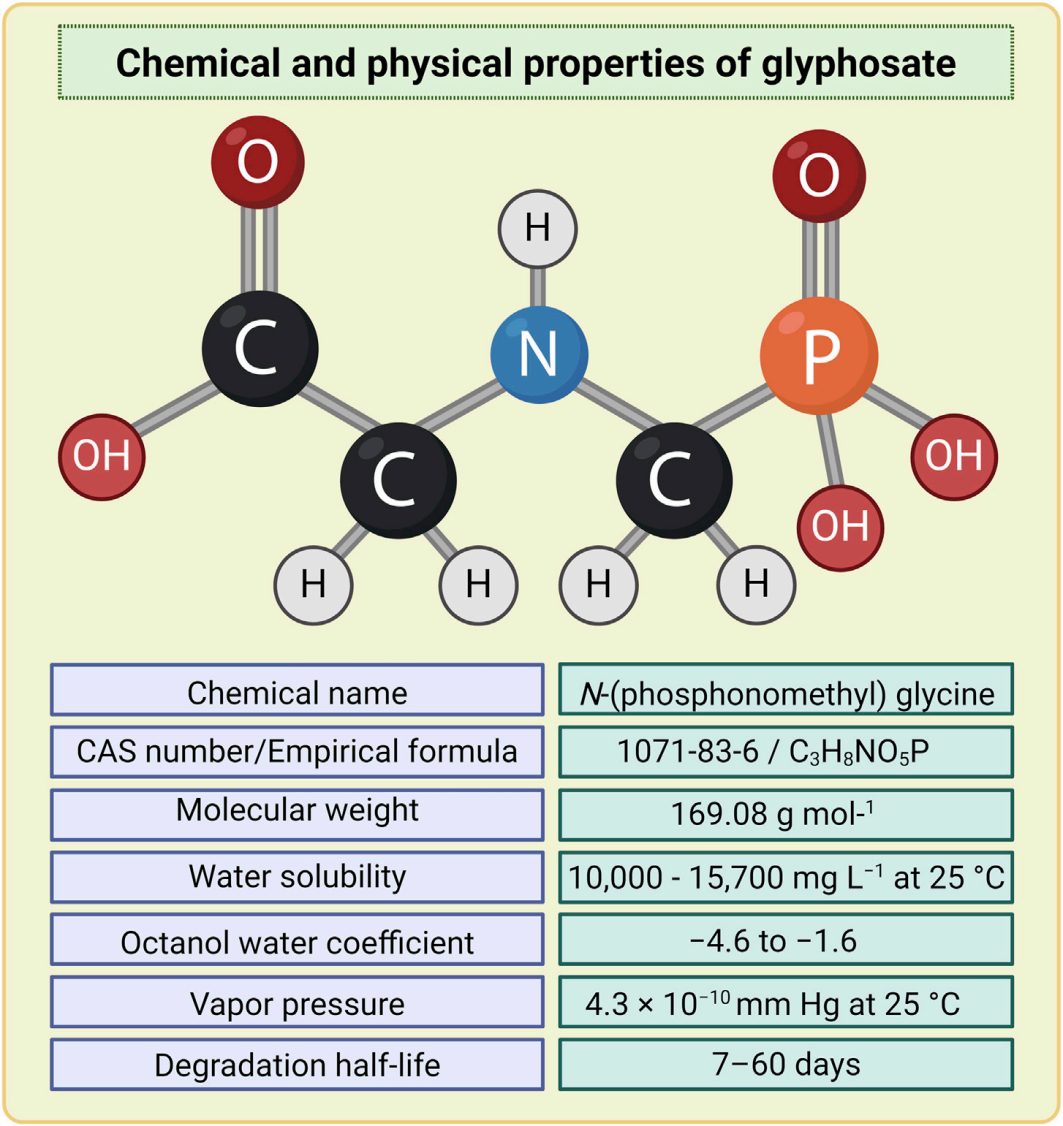
Chemical and physical properties of glyphosate. Image has been created with BioRender.com.

GLY is a white, odourless, and crystalline solid classified as an organophosphorus compound. Its simple chemical structure and its remarkable stability under a variety of reaction conditions (e.g., pH, temperature, etc.) has allowed it to be manufactured in different synthesis routes. A frequently adopted synthetic pathways is the one based on the multi-step reaction between glycine, formaldehyde and DMP (dimethyl phosphite) leading to the product GLY with high yields, via several non-isolatable intermediates (Kanissery et al., 2019; Kim et al., 2019; Munoz et al., 2021). Formally can be synthesized by oxidative coupling of the methyl group of methylphosphonic acid with the amine group of glycine (Kanissery et al., 2019; Kim et al., 2019; Munoz et al., 2021). GLY can be considered as a phosphonomethyl derivative of the natural amino acid glycine (Lanzarin Gab et al., 2023). Indeed, GLY is an amphoteric chemical which presents three functional groups characterizing the molecule: carboxylic, amine and phosphonic. As a result, similar to amino acids, GLY is able to form a zwitterionic structure (Fliss et al., 2021). This is reflected in its high polarity and excellent solubility in water and very low lipophilicity (Lanzarin Gab et al., 2023) (Figure 3). Moreover, GLY is photochemically non-degradable, stable in air and is a non-volatile substance undergoing rapid inactivation in soil through adsorption and degradation (John et al., 1988; Kanissery et al., 2019). The mode of action of GLY is to inhibit the activity of the enzyme EPSPS, which is required in the shikimate pathway. Inhibition of EPSPS prevents the synthesis of AAAs such as tyrosine, phenylalanine and alanine in plants, which results in their subsequent death (Kanissery et al., 2019; Marino et al., 2021; Munoz et al., 2021; Ferrante et al., 2023) (Figure 1). GLY appears to be quite resistant to degradation due to the presence of the stable carbon-phosphate bond (Singh et al., 2020). Despite this, GLY can undergo biodegradation through microbial action, both in soil and water, by breaking the carbon-nitrogen (C-N) bond (Noori et al., 2018; Panzacchi et al., 2018). The biodegradation of GLY occurs by action of the enzyme GLY oxidoreductase, through two main metabolic pathways. The first leads to the formation of AMPA (major microbial metabolite) and glyoxylate; the latter causes the conversion of GLY to glycine (Munoz et al., 2021). GLY has a lower toxicity compared to other GBHs, which contain additional ingredients in the commercial formulations in order to modify certain properties of GLY, increasing its penetration into plants and leading to a significant enhancement of GLY’s toxicity (Marino et al., 2021). For instance, Roundup^®^ contains in its formulation numerous co-formulants, one of these is polyetholoxylated tallow amine (POEA) employed as a surfactant in GBHs. In numerous studies, POEA has been shown to be amongst the surfactants that exhibit the greatest toxic effects (Benachour and Seralini, 2009; Peillex and Pelletier, 2020; Miko and Hettyey, 2023). As mentioned above, GLY is an analogue of glycine, which is the simplest among the amino acids. Glycine results to be crucial in synthesis of numerous proteins and exhibits essential singular properties, such as the ability to bind both plasma membrane and cytoskeleton (Perez-Torres et al., 2017). Due to GLY’s structural similarity to glycine, the latter is erroneously misled and substituted with GLY, which is incorporated into peptides in different processes of protein synthesis; leading to numerous damages including hypothyroidism, Alzheimer’s disease and kidney failure (Samsel and Seneff, 2016). However, in a recent work it was demonstrated, through a molecular modelling approach, how GLY is unable to bind to the glyci-tRNA synthetase active site, due to the steric hindrance caused by the phosphate group, that result absent in the glycine (Antoniou et al., 2019). In support of this thesis, in the same paper, a proteomic study was carried out involving mass spectrometry analysis, which enables a distinction between the different molecular weights, and therefore also capable of discriminating if GLY is or not incorporated into proteins. Statistical analysis of the overall proteome shows that there are no substantial differences between GLY-treated and untreated samples, leading to the conclusion that GLY is unlikely to be able to binding to proteins. Therefore, it is clear that there is a controversy in literature regarding the consequences of the chemical similarity of GLY and glycine.

## 4 Glyphosate and human exposure routes

**FIGURE 4:**
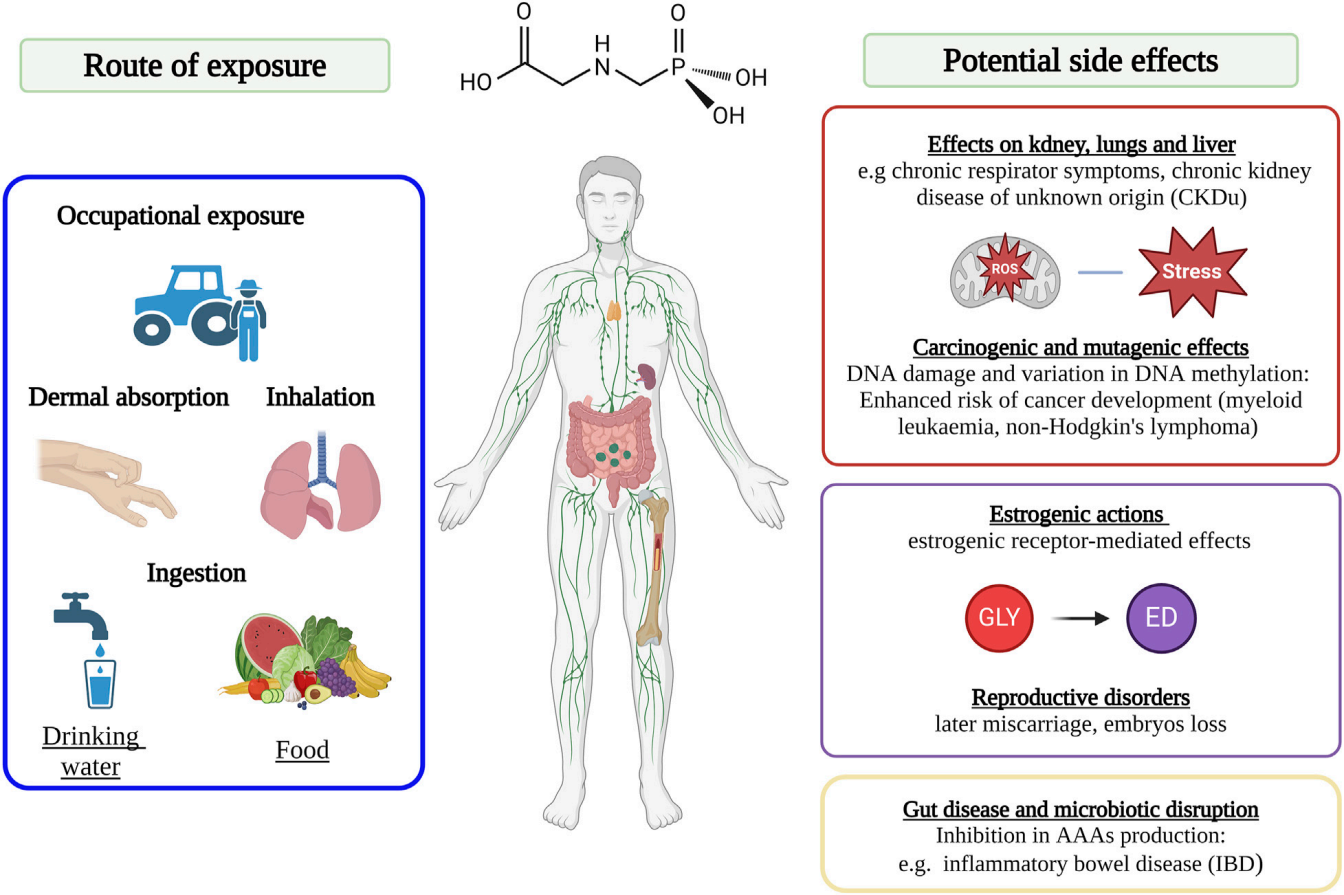
Scheme of the main exposure and contamination routes of glyphosate’s and glyphosate-based herbicide’s (GBHs) in humans. Image has been created with BioRender.com.

GLY is able to enter human bodies through different exposure routes, among which the most notable include: dermal absorption, inhalation, ingestion as well as intake of GLY-contaminated foodstuffs (Figure 4). In several studies, it was observed that when GLY reaches the human body, it tends to accumulate in kidneys, liver and colon (Torretta et al., 2018; Marino et al., 2021). The molecule of GLY is eliminated via both urine and faeces, as an unmodified compound in greater quantities in comparison to its main metabolite AMPA (Williams et al., 2000; Panzacchi et al., 2018; Peillex and Pelletier, 2020; Leblanc et al., 2024). In particular, GLY can be found in high amounts in workers’ urine, but it may be detected in other biological fluids, such as breast milk and blood, with an incidence rate in general population of approximately 60%–80%, including children as well, demonstrating how exposure occurs not only from work-related origin (Torretta et al., 2018; Van Bruggen et al., 2018; Connolly et al., 2019). In literature itself there are inconsistencies concerning the routes of exposure of GLY and the resulting impact. Indeed, even if many authors suggest that dermal absorption represents the primary route of GLY human exposure, several in *in vivo* and in *in vitro* studies have indicated how skin absorption may be regarded as negligible (Williams et al., 2000; Connolly et al., 2020; Pierce et al., 2020; Sidthilaw et al., 2022). Indeed, several studies conducted on rabbits have indicated how GLY is extremely eye-irritant, but only slightly irritant to skin (Shin et al., 2020; Ferrante et al., 2023). However, several publications have pointed out how GBH products are likely to induce severe chemical burns (Mariager et al., 2013; Shin et al., 2020), and evidence shows that GLY’s epidermic absorption capability is 5x higher if exposed to damaged skin as compared to the healthy (Heu et al., 2012; Shin et al., 2020). Indeed, the only documented death due to skin exposure to GLY involves an 81-year-old Korean man, who did not wash his skin for more than 48 h following the use of an herbicide containing GLY, which had previously caused him severe skin lesions (Shin et al., 2020). Regarding occupational exposure, especially for farmers, the most important route is through the inhalation of GBH products present in aerosol, vapour or dust form (Damalas and Koutroubas, 2016). Further, it has been recently reported that agricultural chemicals, such as GBH, can travel with farm dust into nearby cities, exacerbating the exposure risks (Miousse et al., 2023). Such exposure is particularly harmful, as it could lead to chronic respiratory symptoms and decline of lung function (Tarmure et al., 2020; Pandher et al., 2023). The principal breathing pathology associated with GLY air exposure is a specific atopic asthma, known as ‘wheezing’ (Ye et al., 2013). Furthermore, in other studies it was found that the inhalation of GLY in combination with other substances (Pandher et al., 2021a; b), for example, with lipopolysaccharide (LPS), which is a constituent of the external membrane of Gram-negative bacteria, frequently present in soil and inhalable through dust (Zielen et al., 2015), caused worse human health effects than those triggered by individual exposures. In this regard, attention can be drawn to studies conducted by Pandher and colleagues (Pandher et al., 2021b), showing that the inhalation of air particles made up of both LPS and GLY, caused more serious pulmonary inflammation as compared to inhalation of two individual compounds. Finally, the exposure can also occur through intake of GLY-contaminated foods and this route of exposure became increasingly alarming throughout the years, due to global overuse of GHBs (Myers et al., 2016; Rawat et al., 2023). Indeed, due to its high stability, GLY is able to accumulate both in treated crops and in different environmental compartments, such as soil and water (Martins-Gomes et al., 2022). Therefore, the widespread environment presence of GLY also leads to a diffuse contamination of plant-based foodstuffs (Gillezeau et al., 2019; Narimani and da Silva, 2020). In addition to the above, crops are repeatedly treated with GBHs during each season since such products are actually not only used as herbicides, but also frequently applied as crop-drying agents in cereal harvesting (Van Bruggen et al., 2018; Marino et al., 2021). As a result, GLY is also diffusely detected in foodstuffs like cereals, grains, and fruits (Torretta et al., 2018; Kanissery et al., 2019). Fodder crops are also routinely treated with GLY products. The outcome is that GLY has been found in significant amounts both in the urine of cows and in the meat of cattle (Feltracco et al., 2022). In the available literature data, one of the biggest inconsistencies appears to be that the majority of GLY levels detected in food are below the acceptable thresholds and are scarcely ever detected in milk, meat and fish (Kolakowski et al., 2020; Munoz et al., 2021). Instead, as mentioned above, several studies have actually found that GLY is strongly present in environment and general population is daily exposed to it via several routes, including consumption of plant-based foods. From the foregoing, it can be assumed that daily exposure to this herbicide could be harmful to humans.

### 4.1 Effects of glyphosate exposure on liver, kidney and lungs

Oxidative stress is a phenomenon which causes an alteration in the equilibrium between production of reactive oxygen species (ROS) and organism’s activated anti-oxidant systems. This imbalance may lead to several issues, including damage of lipids and proteins, activation of apoptotic pathways and/or the initiation of inflammatory phenomena in both tissues and cells (Pizzino et al., 2017; Vona et al., 2021). Although natural cellular physiological processes trigger the formation of ROS (Bardaweel et al., 2018), these may also arise through exposure to exogenous substances, including pesticides (Rahal et al., 2014). Oxidative stress has been recognized by IARC as a key factor in carcinogens and in particular, plays a crucial role in the pathogenesis of hematologic cancers (Chang et al., 2023). Several studies demonstrated that GLY has the potential to induce a variety of adverse cellular effects. These include oxidative stress and inflammation in both human cells and *in vivo* models. Unfortunately, there are only limited research data on human population (Martinez et al., 2020; Chang et al., 2023; Coalova et al., 2023; Makame et al., 2023; Pandher et al., 2023). In a study conducted *in vivo*, it was demonstrated how administration of sub-lethal concentrations of Roundup^®^, or as-is GLY, may induce an oxidative stress response (El-Shenawy, 2009). In fact, such administration leads to considerable glutathione (GSH) depletion and the induction of hepatic tissue oxidative stress, mediated by high levels of lipid peroxidation. Additionally, it also causes an increase in both nitric oxide (NO) concentration and tumour necrosis factor α (TNF-α) level, highlighting their action mechanisms as antioxidant agent destroyers (El-Shenawy, 2009). Moreover, in different *in vitro* studies conducted on human liver cell lines, it was demonstrated that both Roundup^®^ and GLY would induce cytotoxicity, ROS formation, and apoptosis at doses well below the concentrations employed in agricultural applications (Unlu Endirlik et al., 2022; Mehtiyev et al., 2023). Indeed, in the above-mentioned studies, it is shown that ROS formation may cause remarkable modifications in the gene expression of Keap1, L-FABP, Nrf2, HO-1 and HSP70, which are related to the MAPK/ERK pathway (Unlu Endirlik et al., 2022; Mehtiyev et al., 2023). Recent studies have shown that GBHs, as well as GLY alone, may adversely affect kidney functions, resulting in increased blood urea levels (El-Shenawy, 2009; Gunatilake et al., 2019; Kimura et al., 2020). Gunatilahe and colleagues showed that being exposed to contaminated drinking water is associated to high rates of Chronic Kidney Disease of Unknown Origin (CKDu) (Gunatilake et al., 2019). Indeed, GLY has a crucial role in the transport of heavy metals into the kidneys. In farmers occupationally exposed to GLY, the levels of some commonly marker used for the assessment of kidney function have been found altered, amongst which we can mention: urinary neutrophil gelatinase-associated lipocalin (uNGAL), serum creatinine (sCr), urinary microalbumin-to-creatinine ratio (ACR), serum cystatin C (sCys-C), estimated glomerular filtration rate (eGFR), and exposure intensity index (EII) (Mueangkhiao et al., 2020). While, in an *in vivo* study in rats, in which animals were administered with water containing Roundup^®^ at concentrations of 0.1 ppb (equivalent to 0.05 μg/L GLY), both liver and kidney damage were observed (El-Shenawy, 2009). As a matter of fact, such exposures resulted in anatomical signs of disease and alterations in specific blood and urine parameters, markers of hepatic and renal insufficiency (El-Shenawy, 2009). These alterations were likewise confirmed at molecular level by analysing gene expression profiles in both liver and kidney, exhibiting altered expression patterns, which are typical of dysfunctional mitochondrialas pathologies, such as fibrosis, necrosis, and ischemia (El-Shenawy, 2009). Furthermore, Roundup^®^, in an *in vivo* experiment performed at increasing product concentrations (from 5 to 250 mg/kg bw), has also shown detrimental effects on adipose tissue and liver, which can be related to the onset of steatosis and non-alcoholic fatty liver. Indeed, these are the principal organs playing a crucial function in the maintenance of homeostatic energy (Arango Duque and Descoteaux, 2014; Pandey et al., 2019). At the same treatment concentrations, was also observed a significant overexpression of some inflammatory markers’ levels, such as interleukins IL-1β, IL-6, and TNF-α (Pandey et al., 2019; Marino et al., 2021). Work-related GLY exposure may also be regarded as a health risk factor, due to the occurrence of asthma symptoms. Indeed, experiments were conducted in which rats inhaled air samples collected from farms, where GLY was frequently used (Kumar et al., 2014). These studies showed how pulmonary exposure of animals treated with air samples rich in GLY, revealed an increase in eosinophil and neutrophil counts with mast cell degranulation, production of IL-33, thymic stromal lymphopoietin (TSLP), IL-13 and IL-5 (Kumar et al., 2014). In addition, lung inflammatory processes arising from GLY exposure were also observed in Pandher and colleagues in *in vivo* study (Pandher et al., 2023). In this study, rats were treated with 1 µg doses of GLY and an enhancement of neutrophil count levels, myeloperoxidase, TNF-α, IL-6, KC, ICAM-1 and TLR-2 expression was observed. The increase of inflammatory cytokines, in particularly the cytokine IL-33 and TSLP, intensifies the inflammatory reaction leading to bronchial hyperactivity. This suggests that GLY exposure may cause disruption of the airway epithelial barrier.

### 4.2 Glyphosate exposure a possible cause of gut disease and microbiotic disruption

GLY can be regarded as a significant environmental factor in the development of diseases associated with dysbiosis of the gut microbiota, like inflammatory bowel disease (IBD) (Chang et al., 2023; Lehman et al., 2023). The status of gut microbiota, which has the function of maintaining intestinal health by promoting digestion, synthesizing vitamins, and supporting the immune system, may be affected by exposure to exogenous chemicals, contributing to the development of various clinical conditions (e.g., Chron’s disease and metabolic disorders) (Mesnage et al., 2019). In fact, the gut represents one of the main barriers for the organism to be exposed to exogenous substances and is specifically vulnerable to exposure to pesticides, including GLY (Abou Diwan et al., 2023). Since the human body naturally does not produce some essential AAAs, such as tryptophan, tyrosine or phenylalanine, these need to be taken from the diet and/or produced by gut microbes (Lopez and Mohiuddin, 2024). Exposure to GLY could leads to an alteration in microbiota structure through interaction with the shikimate pathway, resulting in an inhibition in AAAs production (Mesnage and Antoniou, 2020). Moreover, the GLY disruption of the shikimate route leads to a decrease in plant levels of these nutrients and, as a consequence, in their bioavailability to living beings (Zobiole et al., 2010). As previously outlined, alteration of microbiota could potentially induce various gastrointestinal disorders, including IBD and irritable bowel syndrome (IBS). In addition to the above, a study conducted on rats suggested that exposure to GLY can lead to inflammatory response at small intestine level. Indeed, GLY affected the gut microbial composition of these rats, causing a significant reduction in *Lactobacillus* (Tang et al., 2020) and also a disruption of intestinal mucosal function, inducing dysbiosis and chronic inflammation (Huang et al., 2020; Fu et al., 2021; Meng et al., 2022). To support the foregoing, an *in vivo* study concerning inflammatory bowel disease should be mentioned, in which it was reported how GLY exposure would also lead to a significant upregulation of C-type lectin-like receptor 2 (CLEC-2). Such lectin-like receptor, expressed by both immune cells and platelets, is likely to be implicated in cancer development, as well as being related to the increase in enter pathogenic infections (Barnett et al., 2020). In addition, further evidences indicate how the destruction of tight junctions between intestinal and colonic epithelium cells, triggered by GLY exposure, is liable to induce the permeable bowel syndrome (Qiu et al., 2020). Furthermore, the dysbiosis and microbiota imbalance caused by GLY exposure could lead to the initiation of pro-inflammatory cascades in central nervous system (CNS), mediated by lipopolysaccharide (LPS). Through this process, the permeability of the blood brain barrier increases, causing an impairment of specific cognitive functions (Rueda-Ruzafa et al., 2019; Izumi et al., 2024).

### 4.3 Glyphosate and its possible carcinogenic and/or mutagenic effects

Biotransformation processes undergone by xenobiotics, including GLY, in some cases lead to ROS production, which is liable to cause DNA damage. Such injury has the potential to induce mutations and out-of-control cell proliferation, resulting occasionally in the development of cancer. In recent years, GLY’s potential genotoxic effects became a topic of intense debate, both inside scientific community as well as amongst the various international agencies (Tarazona et al., 2017; Nagy et al., 2019). The scientific divergences behind the debate, may arise both from the use of different data sets, and from dissimilar methodologies for interpreting ambiguous results. For example, the IARC considered sufficient data supplied by case-control researches, unconfirmed by cohort studies, to conclude the evidence of correlation between GLY exposure and non-Hodgkin’s lymphoma. EFSA re-evaluated the same information, giving more weight to the absence of epidemiological studies, considering insufficient for classification (Tarazona et al., 2017). These discussions have become more heated following the decision to renew GLY’s authorization at EU level for another 10 years. The discrepancies observed among the various studies conducted to determine DNA damage caused by GLY exposure, are due to several reasons. The most relevant are related to the different experimental approaches employed and either for the choice of testing GLY at high concentrations and in presence of co-formulants, not considering GLY on its own. In addition, the available studies have been conducted using old GBH formulations, containing the notorious tallow amines. These co-formulants were widely utilized for their efficacy, but exhibited a detrimental toxicological profile (Martens et al., 2019), for which reason they were banned in 2016 at European level. In recent studies conducted *in vitro*, GLY showed genotoxic effects at both cellular and genomic levels, in several cell lines, but especially in peripheral blood mononuclear cells (PBMCs) (Nagy et al., 2021). Supporting this, in Andreotti and colleagues’ epidemiological study, a correlation was observed between the GLY use and the increased risk of developing acute myeloid leukaemia (AML) (Andreotti et al., 2018). Furthermore, in *in vitro* studies conducted on human lymphocytes exposed to a GLY concentration of 0.5 μg/mL, corresponding to the EFSA established Acceptable Daily Intake (ADI) (EFSA, 2015), it has been observed how GLY potentially induces DNA damage, including DNA aberrations, chromosome breaks, chromatids, dicentric chromosomes, rings and acentric fragments, and induce micronuclei (MNi). These harms suggest how GLY exposure potentially leads to an increased risk of developing cancer in the exposed people (Santovito et al., 2018; Nagy et al., 2021). The potential genotoxic effect was assessed *in vitro,* not only by considering GLY, but also evaluating the impacts induced by Roundup^®^ formulation and AMPA metabolite. Findings from these studies indicated that all three compounds might indifferently induce single-strand breaks. Furthermore, it was observed how both GLY and Roundup^®^ under investigation in comet assay, respectively at concentrations of 1,000 and 10 μM, cause similar DNA damage (Wozniak et al., 2018). Such DNA lesions are caused by oxidative stress, triggering the production of 8-oxodG, which can promote incorporation of adenine in place of cytosine, resulting in guanine:cytosine → thymine:adenine mutation (Wozniak et al., 2018). Furthermore, GLY is able to induce changes in DNA methylation. In fact, in PBMCs treated with high concentrations of GLY (from 10 to 100 μM), a state of DNA hypo methylation was found, whereas in particular at lower concentrations (0.5 μM) GLY is able to induce an induced in hypo-methylation promoter of P21 and a hyper-methylation promoter of TP53, which are involved in the activation of apoptotic pathways (Wozniak et al., 2020). Alterations in methylation, both at the general level and in promoter regions of genes involved in various cellular processes, are recognized as likely responsible for the enhanced risk of cancer development (Ehrlich, 2019; Wozniak et al., 2020). From the above, it is possible to conclude that GLY may induce epigenetic alterations that can disrupt the normal methylation processes and gene expression in human PBMCs, resulting in cellular transformation. Due to such scientific evidence, IARC concluded that an association between GLY exposure and the development of non-Hodgkin’s lymphoma is possible (IARC, 2017). The Genotoxic effects of GLY and GHBs were also observed in human liver cells (HepG2). In Gasnier et al. (2009), human liver cell lines were treated with both GLY and also with different Roundup^®^ formulations containing increasing amounts of active ingredient (from 7.2 to 450 g/L) that induced both DNA damage and anti-estrogenic activity on estrogen nuclear receptors (nERs), ERα and ERβ. Similar results were also obtained in a research conducted on the human buccal epithelial cell line (TR146) demonstrating, by comet assay, how GLY is able to cause DNA damage resulting in probable single-strand breaks, apurinic sites, MNi and nuclease buds (NB) (Koller et al., 2012). In addition, both GLYs and GBHs have been found to promote the maintenance of tumour environment leading to the development of resistance to chemotherapeutics employed in treatment of certain cancer forms. For example, in human glioblastoma cell lines, it was observed an enhancement of all biomarkers (GST, P-gp/ABC, and MRP) involved in the mechanism of multidrug resistance (MDR); that promoting oxidative stress and an increased resistance to apoptosis (Doganlar et al., 2020).

### 4.4 Estrogenic effects of glyphosate and glyphosate-based herbicides

Several studies have demonstrated how both GLYs and GBHs can be regarded as endocrine disruptors (EDs), as they are able to induce estrogenic or androgenic nuclear receptors-mediated effects (Mesnage et al., 2017; Munoz et al., 2021). In a research study conducted *in vitro* on T47D hormone-dependent and MDA-MB231 hormone-independent human breast carcinoma cell lines, it was demonstrated how GLY exposure possibly induces estrogen-responsive element (ERE) transcriptional activity, with consequent induction of cell proliferation in hormone-dependent carcinoma lines. By contrast, in hormone-independent cells the same activity was not observed (Thongprakaisang et al., 2013). The same authors also demonstrated that the GLY proliferative effect is nullified following exposure to an ER inhibitor drug (ICI 182780), confirming the estrogenic GLY activity (Thongprakaisang et al., 2013). In recent *in vitro* experiments carried out in human endometrial (nERs-positive) and breast cancer (nERs-negative) cell lines, genotoxic effects induced by GLY have been observed in relatively low concentrations (75–500 μg/mL) and after short-time treatment. It is interesting to highlight, how toxic effects have been observed on both cell lines, but with a greater influence on the ER-positive endometrial lines. Therefore, from the above findings, it can be shown that genotoxic effects caused by both GLYs and GBHs, can also be attributed to nERs status (De Almeida et al., 2018). Indeed, as the breast cancer lines are hormone-independent, they express low levels of nERs. Consequently, in order for GLY to exert its effect, it should act through non-estrogenic mechanisms. As the interaction between nERα and GLY has proven to be weak, the genetic activation is plausibly mediated by a non-binding mechanism (Mesnage et al., 2017). In a recent work involving the Ishikawa cell line derived from human endometrial carcinoma, GLY was shown to induce E2 (17β-estradiol)-like effects on the epithelial-mesenchymal transition process. These influences are manifested through promoting the migration and cell invasion, and by down regulating of E-cadherin mRNA expression, in a similar mode as occurs with E2. This behavior has been reversed following treatment with the drug ICI 182780 (Gastiazoro et al., 2020). Hence, there is evidence from these studies that both GLY and GBHs, exhibit estrogenic properties, although these are milder than the ones observed in E2. However, data in the literature are lacking, so further research is needed to compare the estrogenic potency of GLY and GHBs with those of E2 and other estrogen-like EDs, such as phthalates. Additionally, GBHs has been shown to reduce aromatase enzyme activity in human placental cells (JEG3) by decreasing its gene expression. This process occurs at concentrations below of those employed for agricultural purposes (Richard et al., 2005). During the same research over mentioned, this inhibiting effect of the aromatase enzyme, triggered by both GLY and GHBs, was also observed in equine and human testicular microsomes, proving an interaction with the purified enzyme’s active site (Richard et al., 2005). The before-mentioned data are corroborated in a study conducted by Benachour et al. (2007) which revealed that both GBHs and GLY are able to inhibit the activity of the enzyme in both HEK 293 and JEG3 cells, which are derived from human placenta or its extracts, as well as from mammalian testis. In spite of the fact that the GBHs represent the most widely used pesticides worldwide, relatively few human epidemiological studies concerning fertility are available. In a Canadian study in Ontario population, it was found that pre-conception GLY exposure, could be responsible for increased risk of later miscarriage (Arbuckle et al., 2001). While, in another more recent research, conducted on a U.S. Indiana cohort, it is reported that urinary levels showing higher GLY concentration, might be associated to a shorter gestation period (Parvez et al., 2018). Finally, in a Thailand study, high GLY levels were detected in fetal and maternal serum of gestating women, engaged in agricultural labor or living in farming households (Kongtip et al., 2017). However, additional epidemiological investigations are necessary to provide further understanding of potential adverse fertility effects induced by GLY and GBHs. In contrast, several existing animal studies are available demonstrating the harmful effects of GLY and GBHs on the reproductive health at environmentally relevant doses. Among the worst effects, we can cite embryos loss prior to and post-implantation, delay in growth, as well as congenital abnormalities (Milesi et al., 2018; Milesi et al., 2019; Pham et al., 2019; Milesi et al., 2021).

## 5 Conclusion

The exposure to pesticides is well known to cause serious damage to both environment and human health. Several scientific studies demonstrated how being exposed to GLY and GBHs, may lead to the occurrence of various conditions in humans, like the onset of inflammatory diseases, neurological disorders and reproductive disruptions (Corti et al., 2022; Tassinari et al., 2023). The molecular mechanisms causing these observed effects are not completely understood. Part of the origin of these detrimental effects, could be associated with the structural similarity of GLY to glycine, which is confused and substituted with GLY in different processes of protein synthesis, causing both neurotoxic and cytotoxic effects. However, a discordance among the literature data exists, as it was also demonstrated through molecular modelling approach and proteomic analysis, that GLY is unlikely to bind proteins, due to its steric hindrance. These discordances suggest that the consequences of GLY’s similarity to glycine are still an open issue. This process is just one of many that would explain the harmful impacts caused by GLY. Indeed, both regulatory agencies and scientists differ in their opinions concerning the assessment of the health risks induced by GLY and GBHs exposure. Recently, at EU level, the use of GLY has been renewed for another 10 years, despite evidence of genotoxic and mutagenic effects from several *in vitro* studies. These experiments have been conducted either with high doses of glyphosate as well as in presence of co-formulants (e.g., tallow amines), which are considered by the competent authorities to be the most responsible for the negative human health effects caused by GHBs. In order to exclude the potential carcinogenic and genotoxic effects of GLY, the studies should be conducted by using glyphosate alone rather than in formulation and in concentrations below, or equivalent, to the exposure doses considered safe for human health. Additionally, genotoxicity tests should be performed in *in vivo* studies with low doses active substance alone. Therefore, the negative human health aspects arising from GLY exposure, still remains an open topic to be investigated, particularly considering the recent European-level renewal of the active substance for another 10 years.

## References

[B1] Abou Diwan M. Bach V. Gosselet F. Khorsi-Cauet H. Candela P. (2023). Impact of pesticide residues on the gut-microbiota–blood–brain barrier Axis: a narrative review. Int. J. Mol. Sci. 24, 6147. 10.3390/ijms24076147 37047120 PMC10094680

[B2] Abrams S. A. Albin J. L. Landrigan P. J. Committee On N. Council On Environmental H. Climate C. (2024). Use of genetically modified organism (GMO)-Containing food products in children. Pediatrics 153 (1), e2023064774. 10.1542/peds.2023-064774 38073334

[B3] Acquavella J. (2023). Epidemiologic studies of glyphosate and non-Hodgkin's lymphoma: a review with consideration of exposure frequency, systemic dose, and study quality. Glob. Epidemiol. 5, 100101. 10.1016/j.gloepi.2023.100101 37638378 PMC10445963

[B4] Altmanninger A. Brandmaier V. Spangl B. Gruber E. Takács E. Mörtl M. Klátyik S. (2023). Glyphosate-based herbicide formulations and their relevant active ingredients affect soil springtails even five months after application. Agriculture 13, 2260. 10.3390/agriculture13122260

[B5] Andreotti G. Koutros S. Hofmann J. N. Sandler D. P. Lubin J. H. Lynch C. F. (2018). Glyphosate use and cancer incidence in the agricultural health study. J. Natl. Cancer Inst. 110 (5), 509–516. 10.1093/jnci/djx233 29136183 PMC6279255

[B6] Annett R. Habibi H. R. Hontela A. (2014). Impact of glyphosate and glyphosate-based herbicides on the freshwater environment. J. Appl. Toxicol. 34 (5), 458–479. 10.1002/jat.2997 24615870

[B7] Antoniou M. N. Nicolas A. Mesnage R. Biserni M. Rao F. V. Martin C. V. (2019). Glyphosate does not substitute for glycine in proteins of actively dividing mammalian cells. BMC Res. Notes 12 (1), 494. 10.1186/s13104-019-4534-3 31395095 PMC6686468

[B8] Arango Duque G. Descoteaux A. (2014). Macrophage cytokines: involvement in immunity and infectious diseases. Front. Immunol. 5, 491. 10.3389/fimmu.2014.00491 25339958 PMC4188125

[B9] Arbuckle T. E. Lin Z. Mery L. S. (2001). An exploratory analysis of the effect of pesticide exposure on the risk of spontaneous abortion in an Ontario farm population. Environ. Health Perspect. 109 (8), 851–857. 10.1289/ehp.01109851 11564623 PMC1240415

[B10] Bardaweel S. K. Gul M. Alzweiri M. Ishaqat A. Ha A. L. Bashatwah R. M. (2018). Reactive oxygen species: the dual role in physiological and pathological conditions of the human body. Eurasian J. Med. 50 (3), 193–201. 10.5152/eurasianjmed.2018.17397 30515042 PMC6263229

[B11] Barnett J. A. Quin C. S. Bonnie R. La Berge A. Alejandra Verdugo Meza A. Hart M. (2020). 216 EXAMINING THE EFFECTS OF GLYPHOSATE EXPOSURE ON THE GUT BACTERIOME AND INFLAMMATION IN A MURINE MODEL OF COLITIS. J. Can. Assoc. Gastroenterol. 3, 89–90. 10.1093/jcag/gwz047.215

[B12] Battaglin W. A. Meyer M. T. Kuivil a K. M. Dietz e J. E. (2014). Glyphosate and its degradation product AMPA occur frequently and widely in U.S. Soils, surface water, groundwater, and precipitation. J. Am. Water Resour. Assoc. 50 (2), 275–290. 10.1111/jawr.12159

[B13] Benachour N. Seralini G. E. (2009). Glyphosate formulations induce apoptosis and necrosis in human umbilical, embryonic, and placental cells. Chem. Res. Toxicol. 22 (1), 97–105. 10.1021/tx800218n 19105591

[B14] Benachour N. Sipahutar H. Moslemi S. Gasnier C. Travert C. Seralini G. E. (2007). Time- and dose-dependent effects of roundup on human embryonic and placental cells. Arch. Environ. Contam. Toxicol. 53 (1), 126–133. 10.1007/s00244-006-0154-8 17486286

[B15] Benbrook C. M. (2016). Trends in glyphosate herbicide use in the United States and globally. Environ. Sci. Eur. 28 (1), 3. 10.1186/s12302-016-0070-0 27752438 PMC5044953

[B16] Brookes G. Taheripour F. Tyner W. E. (2017). The contribution of glyphosate to agriculture and potential impact of restrictions on use at the global level. Gm. Crops Food 8 (4), 216–228. 10.1080/21645698.2017.1390637 29035143 PMC5790413

[B17] Bukowska B. Wozniak E. Sicinska P. Mokra K. Michalowicz J. (2022). Glyphosate disturbs various epigenetic processes *in vitro* and *in vivo* - a mini review. Sci. Total Environ. 851 (Pt 2), 158259. 10.1016/j.scitotenv.2022.158259 36030868

[B18] Cellier M. Anthony N. Bruneau C. Descatha A. (2022). Determination of glyphosate and AMPA in blood can predict the severity of acute glyphosate herbicide poisoning. Lab. Med. 53 (4), 394–398. 10.1093/labmed/lmac002 35150270

[B19] Chang V. C. Andreotti G. Ospina M. Parks C. G. Liu D. Shearer J. J. (2023). Glyphosate exposure and urinary oxidative stress biomarkers in the Agricultural Health Study. J. Natl. Cancer Inst. 115 (4), 394–404. 10.1093/jnci/djac242 36629488 PMC10086635

[B20] Chaufan G. Coalova I. Rios de Molina Mdel C. (2014). Glyphosate commercial formulation causes cytotoxicity, oxidative effects, and apoptosis on human cells: differences with its active ingredient. Int. J. Toxicol. 33 (1), 29–38. 10.1177/1091581813517906 24434723

[B21] Coalova I. March H. Rios de Molina M. D. C. Chaufan G. (2023). Individual and joint effects of glyphosate and cypermethrin formulations on two human cell lines. Toxicol. Appl. Pharmacol. 461, 116398. 10.1016/j.taap.2023.116398 36702315

[B22] Connolly A. Coggins M. A. Galea K. S. Jones K. Kenny L. McGowan P. (2019). Evaluating glyphosate exposure routes and their contribution to total body burden: a study among amenity horticulturalists. Ann. Work Expo. Health 63 (2), 133–147. 10.1093/annweh/wxy104 30608574

[B23] Connolly A. Coggins M. A. Koch H. M. (2020). Human biomonitoring of glyphosate exposures: state-of-the-art and future research challenges. Toxics 8 (3), 60. 10.3390/toxics8030060 32824707 PMC7560361

[B24] Connolly A. Koch H. M. Bury D. Koslitz S. Kolossa-Gehring M. Conrad A. (2022). A human biomonitoring study assessing glyphosate and aminomethylphosphonic acid (AMPA) exposures among farm and non-farm families. Toxics 10 (11), 690. 10.3390/toxics10110690 36422898 PMC9697524

[B25] Corti M. Lorenzetti S. Ubaldi A. Zilli R. Marcoccia D. (2022). Endocrine disruptors and prostate cancer. Int. J. Mol. Sci. 23 (3), 1216. 10.3390/ijms23031216 35163140 PMC8835300

[B26] Damalas C. A. Koutroubas S. D. (2016). Farmers' exposure to pesticides: toxicity types and ways of prevention. Toxics 4 (1), 1. 10.3390/toxics4010001 29051407 PMC5606636

[B27] De Almeida L. K. S. Pletschke B. I. Frost C. L. (2018). Moderate levels of glyphosate and its formulations vary in their cytotoxicity and genotoxicity in a whole blood model and in human cell lines with different estrogen receptor status. 3 Biotech. 8 (10), 438. 10.1007/s13205-018-1464-z PMC6170875 30306007

[B28] Defarge N. Spiroux de Vendomois J. Seralini G. E. (2018). Toxicity of formulants and heavy metals in glyphosate-based herbicides and other pesticides. Toxicol. Rep. 5, 156–163. 10.1016/j.toxrep.2017.12.025 29321978 PMC5756058

[B29] Demonte L. D. Michlig N. Gaggiotti M. Adam C. G. Beldomenico H. R. Repetti M. R. (2018). Determination of glyphosate, AMPA and glufosinate in dairy farm water from Argentina using a simplified UHPLC-MS/MS method. Sci. Total Environ. 645, 34–43. 10.1016/j.scitotenv.2018.06.340 30015116

[B30] Doganlar O. Doganlar Z. B. Kurtdere A. K. Chasan T. Ok E. S. (2020). Chronic exposure of human glioblastoma tumors to low concentrations of a pesticide mixture induced multidrug resistance against chemotherapy agents. Ecotoxicol. Environ. Saf. 202, 110940. 10.1016/j.ecoenv.2020.110940 32800223

[B31] ECHA (2017). Glyphosate not classified as a carcinogen by ECHA. Available at: https://echa.europa.eu/it/-/glyphosate-not-classified-as-a-carcinogen-by-echa.

[B32] EFSA (2011). Submission of scientific peer-reviewed open literature for the approval of pesticide active substances under Regulation (EC) No 1107/2009. EFSA J. 9. 10.2903/j.efsa.2011.2092

[B33] EFSA (2015). Conclusion on the peer review of the pesticide risk assessment of the active substance glyphosate. EFSA J. 13. 10.2903/j.efsa.2015.4302 PMC10369247 37502013

[B34] Ehrlich M. (2019). DNA hypermethylation in disease: mechanisms and clinical relevance. Epigenetics 14 (12), 1141–1163. 10.1080/15592294.2019.1638701 31284823 PMC6791695

[B35] El-Shenawy N. S. (2009). Oxidative stress responses of rats exposed to Roundup and its active ingredient glyphosate. Environ. Toxicol. Pharmacol. 28 (3), 379–385. 10.1016/j.etap.2009.06.001 21784030

[B36] EPA (2020). Available at: https://www.epa.gov.

[B37] EuropeanCommission (2023). Status of glyphosate in the EU. Available at: https://food.ec.europa.eu/plants/pesticides/approval-active-substances-safeners-and-synergists/renewal-approval/glyphosate_en.

[B38] European Food Safety A. Alvarez F. Arena M. Auteri D. Binaglia M. Castoldi A. F. (2023). Peer review of the pesticide risk assessment of the active substance glyphosate. EFSA J. 21 (7), e08164. 10.2903/j.efsa.2023.8164 37502013 PMC10369247

[B39] Feltracco M. Barbaro E. Scopel M. Piazza R. Barbante C. Gambaro A. (2022). Detection of glyphosate residues in feed, saliva, urine and faeces from a cattle farm: a pilot study. Food Addit. Contam. Part A Chem. Anal. Control Expo. Risk Assess. 39 (7), 1248–1254. 10.1080/19440049.2022.2066194 35442859

[B40] Ferrante M. Rapisarda P. Grasso A. Favara C. Oliveri Conti G. (2023). Glyphosate and environmental toxicity with “One Health” approach, a review. Environ. Res. 235, 116678. 10.1016/j.envres.2023.116678 37459948

[B41] Fliss O. Essalah K. Ben Fredj A. (2021). Stabilization of glyphosate zwitterions and conformational/tautomerism mechanism in aqueous solution: insights from *ab initio* and density functional theory-continuum model calculations. Phys. Chem. Chem. Phys. 23 (46), 26306–26323. 10.1039/d1cp03161a 34787605

[B42] Fu Q. Tan Z. Shi L. Xun W. (2021). Resveratrol attenuates diquat-induced oxidative stress by regulating gut microbiota and metabolome characteristics in piglets. Front. Microbiol. 12, 695155. 10.3389/fmicb.2021.695155 34322107 PMC8312259

[B43] Gasnier C. Dumont C. Benachour N. Clair E. Chagnon M. C. Seralini G. E. (2009). Glyphosate-based herbicides are toxic and endocrine disruptors in human cell lines. Toxicology 262 (3), 184–191. 10.1016/j.tox.2009.06.006 19539684

[B44] Gastiazoro M. P. Durando M. Milesi M. M. Lorenz V. Vollmer G. Varayoud J. (2020). Glyphosate induces epithelial mesenchymal transition-related changes in human endometrial Ishikawa cells via estrogen receptor pathway. Mol. Cell Endocrinol. 510, 110841. 10.1016/j.mce.2020.110841 32360565

[B45] Gillezeau C. van Gerwen M. Shaffer R. M. Rana I. Zhang L. Sheppard L. (2019). The evidence of human exposure to glyphosate: a review. Environ. Health 18 (1), 2. 10.1186/s12940-018-0435-5 30612564 PMC6322310

[B46] Grandcoin A. Piel S. Baures E. (2017). AminoMethylPhosphonic acid (AMPA) in natural waters: its sources, behavior and environmental fate. Water Res. 117, 187–197. 10.1016/j.watres.2017.03.055 28391123

[B47] Guilherme S. Santos M. A. Gaivao I. Pacheco M. (2014). DNA and chromosomal damage induced in fish (*Anguilla anguilla* L.) by aminomethylphosphonic acid (AMPA)--the major environmental breakdown product of glyphosate. Environ. Sci. Pollut. Res. Int. 21 (14), 8730–8739. 10.1007/s11356-014-2803-1 24696215

[B48] Gunatilake S. Seneff S. Orlando L. (2019). Glyphosate's synergistic toxicity in combination with other factors as a cause of chronic kidney disease of Unknown origin. Int. J. Environ. Res. Public Health 16 (15), 2734. 10.3390/ijerph16152734 31370256 PMC6695815

[B49] Heu C. Berquand A. Elie-Caille C. Nicod L. (2012). Glyphosate-induced stiffening of HaCaT keratinocytes, a Peak Force Tapping study on living cells. J. Struct. Biol. 178 (1), 1–7. 10.1016/j.jsb.2012.02.007 22369932

[B50] Huang H. M. Pai M. H. Yeh S. L. Hou Y. C. (2020). Dietary exposure to chlorpyrifos inhibits the polarization of regulatory T cells in C57BL/6 mice with dextran sulfate sodium-induced colitis. Arch. Toxicol. 94 (1), 141–150. 10.1007/s00204-019-02615-2 31807802

[B51] IARC (2017). in Some organophosphate insecticides and herbicides. Lyon (FR)). 31829533

[B52] Iohanna Filippi P. F. Grimalt J. O. Butinof M. Amé M. V. Muñoz S. E. Muñoz S. E. (2024). Glyphosate and AMPA in saliva and other traditional human matrices. New findings for less invasive biomonitoring to the exposure to pesticides. Environ. Adv. 15. 100474: 10.1016/j.envadv.2023.100474

[B53] Izumi Y. O'Dell K. A. Zorumski C. F. (2024). Glyphosate as a direct or indirect activator of pro-inflammatory signaling and cognitive impairment. Neural Regen. Res. 19 (10), 2212–2218. 10.4103/1673-5374.391331 38488555 PMC11034589

[B54] Jansons M. Pugajeva I. Bartkevics V. (2018). Occurrence of glyphosate in beer from the Latvian market. Food Addit. Contam. Part A Chem. Anal. Control Expo. Risk Assess. 35 (9), 1767–1775. 10.1080/19440049.2018.1469051 29718772

[B55] John P. Quinn J. M. M. P. R. E. D. Dick R. E. (1988). Glyphosate tolerance and utilization by the microflora of soils treated with the herbicide. Appl. Microbiol. Biotechnol. 29, 511–516v. 10.1007/bf00269078

[B56] Kanissery R. Gairhe B. Kadyampakeni D. Batuman O. Alferez F. (2019). Glyphosate: its environmental persistence and impact on crop health and nutrition. Plants (Basel) 8 (11), 499. 10.3390/plants8110499 31766148 PMC6918143

[B57] Kim J. Leon M. E. Schinasi L. H. Baldi I. Lebailly P. Freeman L. E. B. (2023). Exposure to pesticides and risk of Hodgkin lymphoma in an international consortium of agricultural cohorts (AGRICOH). Cancer Causes Control 34 (11), 995–1003. 10.1007/s10552-023-01748-1 37418114 PMC10533587

[B58] Kim S. Chen J. Cheng T. Gindulyte A. He J. He S. (2019). PubChem 2019 update: improved access to chemical data. Nucleic Acids Res. 47 (D1), D1102-D1109–D1109. 10.1093/nar/gky1033 30371825 PMC6324075

[B59] Kimura T. Yokoyama T. Tanemoto M. (2020). Renal tubular injury by glyphosate-based herbicide. Clin. Exp. Nephrol. 24 (12), 1186. 10.1007/s10157-020-01962-0 32870424

[B60] Kolakowski B. M. Miller L. Murray A. Leclair A. Bietlot H. van de Riet J. M. (2020). Analysis of glyphosate residues in foods from the Canadian retail markets between 2015 and 2017. J. Agric. Food Chem. 68 (18), 5201–5211. 10.1021/acs.jafc.9b07819 32267686

[B61] Koller V. J. Furhacker M. Nersesyan A. Misik M. Eisenbauer M. Knasmueller S. (2012). Cytotoxic and DNA-damaging properties of glyphosate and Roundup in human-derived buccal epithelial cells. Arch. Toxicol. 86 (5), 805–813. 10.1007/s00204-012-0804-8 22331240

[B62] Kongtip P. Nankongnab N. Phupancharoensuk R. Palarach C. Sujirarat D. Sangprasert S. (2017). Glyphosate and paraquat in maternal and fetal serums in Thai women. J. Agromedicine 22 (3), 282–289. 10.1080/1059924X.2017.1319315 28422580

[B63] Kumar S. Khodoun M. Kettleson E. M. McKnight C. Reponen T. Grinshpun S. A. (2014). Glyphosate-rich air samples induce IL-33, TSLP and generate IL-13 dependent airway inflammation. Toxicology 325, 42–51. 10.1016/j.tox.2014.08.008 25172162 PMC4195794

[B64] Landrigan P. J. Belpoggi F. (2018). The need for independent research on the health effects of glyphosate-based herbicides. Environ. Health 17 (1), 51. 10.1186/s12940-018-0392-z 29843729 PMC5972398

[B65] Lanzarin Gab F. L. Fontaínhas-Fernandes A. Monteiro S. M. Venâncio C. Venâncio C. (2023). Effects of glyphosate or glyphosate-based herbicide during the zebrafish life cycle: a review addressing the mechanisms of toxicity. Water 15 (12), 2276. 10.3390/w15122276

[B66] Leblanc P. O. Breton Y. Leveille F. Tessier P. A. Pelletier M. (2024). The impact of the herbicide glyphosate and its metabolites AMPA and MPA on the metabolism and functions of human blood neutrophils and their sex-dependent effects on reactive oxygen species and CXCL8/IL-8 production. Environ. Res. 252 (Pt 1), 118831. 10.1016/j.envres.2024.118831 38580005

[B67] Lehman P. C. Cady N. Ghimire S. Shahi S. K. Shrode R. L. Lehmler H. J. (2023). Low-dose glyphosate exposure alters gut microbiota composition and modulates gut homeostasis. Environ. Toxicol. Pharmacol. 100, 104149. 10.1016/j.etap.2023.104149 37196884 PMC10330715

[B68] Leino L. Tall T. Helander M. Saloniemi I. Saikkonen K. Ruuskanen S. (2021). Classification of the glyphosate target enzyme (5-enolpyruvylshikimate-3-phosphate synthase) for assessing sensitivity of organisms to the herbicide. J. Hazard Mater 408, 124556. 10.1016/j.jhazmat.2020.124556 33243645

[B69] Lopez M. J. Mohiuddin S. S. (2024). “Biochemistry, essential amino acids,” in StatPearls (Treasure Island, FL: StatPearls Publishing). 32496725

[B70] Makame K. R. Ádám B. Nagy K. (2023). Oxidative stress and cytotoxicity induced by Co-formulants of glyphosate-based herbicides in human mononuclear white blood cells. Toxics 11 (976), 976. 10.3390/toxics11120976 38133378 PMC10748038

[B71] Mariager T. P. Madsen P. V. Ebbehoj N. E. Schmidt B. Juhl A. (2013). Severe adverse effects related to dermal exposure to a glyphosate-surfactant herbicide. Clin. Toxicol. (Phila) 51 (2), 111–113. 10.3109/15563650.2013.763951 23360343

[B72] Mária Mörtl G. N. Juracsek J. Darvas B. Kamp L. Rubio F. Székács A. (2013). Determination of glyphosate residues in Hungarian water samples by immunoassay. Microchem. J. 107, 143–151. 10.1016/j.microc.2012.05.021

[B73] Marino M. Mele E. Viggiano A. Nori S. L. Meccariello R. Santoro A. (2021). Pleiotropic outcomes of glyphosate exposure: from organ damage to effects on inflammation, cancer, reproduction and development. Int. J. Mol. Sci. 22 (22), 12606. 10.3390/ijms222212606 34830483 PMC8618927

[B74] Martens M. A. Bleeke M. S. Leopold V. A. Farmer D. R. (2019). Toxicology and human health risk assessment of polyethoxylated tallow amine surfactant used in glyphosate formulations. Regul. Toxicol. Pharmacol. 107, 104347. 10.1016/j.yrtph.2019.03.014 31082430

[B75] Martinez M. A. Rodriguez J. L. Lopez-Torres B. Martinez M. Martinez-Larranaga M. R. Maximiliano J. E. (2020). Use of human neuroblastoma SH-SY5Y cells to evaluate glyphosate-induced effects on oxidative stress, neuronal development and cell death signaling pathways. Environ. Int. 135, 105414. 10.1016/j.envint.2019.105414 31874349

[B76] Martins-Gomes C. Silva T. L. Andreani T. Silva A. M. (2022). Glyphosate vs. Glyphosate-based herbicides exposure: a review on their toxicity. J. Xenobiot. 12 (1), 21–40. 10.3390/jox12010003 35076536 PMC8788447

[B77] Mehtiyev T. Karaman E. F. Ozden S. (2023). Alterations in cell viability, reactive oxygen species production, and modulation of gene expression involved in mitogen-activated protein kinase/extracellular regulating kinase signaling pathway by glyphosate and its commercial formulation in hepatocellular carcinoma cells. Toxicol. Ind. Health 39 (2), 81–93. 10.1177/07482337221149571 36625791

[B78] Meloni F. Satta G. Padoan M. Montagna A. Pilia I. Argiolas A. (2021). Occupational exposure to glyphosate and risk of lymphoma:results of an Italian multicenter case-control study. Environ. Health 20 (1), 49. 10.1186/s12940-021-00729-8 33910586 PMC8082925

[B79] Meng Z. Sun W. Liu W. Wang Y. Jia M. Tian S. (2022). A common fungicide tebuconazole promotes colitis in mice via regulating gut microbiota. Environ. Pollut. 292 (Pt B), 118477. 10.1016/j.envpol.2021.118477 34763016

[B80] Mercurio P. Flores F. Mueller J. F. Carter S. Negri A. P. (2014). Glyphosate persistence in seawater. Mar. Pollut. Bull. 85 (2), 385–390. 10.1016/j.marpolbul.2014.01.021 24467857

[B81] Mesnage R. Antoniou M. N. (2020). Computational modelling provides insight into the effects of glyphosate on the shikimate pathway in the human gut microbiome. Curr. Res. Toxicol. 1, 25–33. 10.1016/j.crtox.2020.04.001 34345834 PMC8320642

[B82] Mesnage R. Benbrook C. Antoniou M. N. (2019). Insight into the confusion over surfactant co-formulants in glyphosate-based herbicides. Food Chem. Toxicol. 128, 137–145. 10.1016/j.fct.2019.03.053 30951798

[B83] Mesnage R. Phedonos A. Biserni M. Arno M. Balu S. Corton J. C. (2017). Evaluation of estrogen receptor alpha activation by glyphosate-based herbicide constituents. Food Chem. Toxicol. 108 (Pt A), 30–42. 10.1016/j.fct.2017.07.025 28711546

[B84] Miko Z. Hettyey A. (2023). Toxicity of POEA-containing glyphosate-based herbicides to amphibians is mainly due to the surfactant, not to the active ingredient. Ecotoxicology 32 (2), 150–159. 10.1007/s10646-023-02626-x 36680666 PMC10008773

[B85] Milesi M. M. Lorenz V. Beldomenico P. M. Vaira S. Varayoud J. Luque E. H. (2019). Response to comments on: perinatal exposure to a glyphosate-based herbicide impairs female reproductive outcomes and induces second-generation adverse effects in Wistar rats. Arch. Toxicol. 93 (12), 3635–3638. 10.1007/s00204-019-02609-0 31720698

[B86] Milesi M. M. Lorenz V. Durando M. Rossetti M. F. Varayoud J. (2021). Glyphosate herbicide: reproductive outcomes and multigenerational effects. Front. Endocrinol. (Lausanne) 12, 672532. 10.3389/fendo.2021.672532 34305812 PMC8293380

[B87] Milesi M. M. Lorenz V. Pacini G. Repetti M. R. Demonte L. D. Varayoud J. (2018). Perinatal exposure to a glyphosate-based herbicide impairs female reproductive outcomes and induces second-generation adverse effects in Wistar rats. Arch. Toxicol. 92 (8), 2629–2643. 10.1007/s00204-018-2236-6 29947892

[B88] Miousse I. R. Hale R. B. Alsbrook S. Boysen G. Broadnax T. Murry C. (2023). Climate change and new challenges for rural communities: particulate matter matters. Sustainability 15 (23), 16192. 10.3390/su152316192 39119507 PMC11307925

[B89] Mueangkhiao P. Siviroj P. Sapbamrer R. Khacha-Ananda S. Lungkaphin A. Seesen M. (2020). Biological variation in kidney injury and kidney function biomarkers among farmers in Lamphun province, Thailand. Environ. Sci. Pollut. Res. Int. 27 (11), 12386–12394. 10.1007/s11356-020-07661-3 31989504

[B90] Munoz J. P. Bleak T. C. Calaf G. M. (2021). Glyphosate and the key characteristics of an endocrine disruptor: a review. Chemosphere 270, 128619. 10.1016/j.chemosphere.2020.128619 33131751

[B91] Myers J. P. Antoniou M. N. Blumberg B. Carroll L. Colborn T. Everett L. G. (2016). Concerns over use of glyphosate-based herbicides and risks associated with exposures: a consensus statement. Environ. Health 15, 19. 10.1186/s12940-016-0117-0 26883814 PMC4756530

[B92] Nagy K. Argaw Tessema R. Szasz I. Smeirat T. Al Rajo A. Adam B. (2021). Micronucleus Formation induced by glyphosate and glyphosate-based herbicides in human peripheral white blood cells. Front. Public Health 9, 639143. 10.3389/fpubh.2021.639143 34109144 PMC8180907

[B93] Nagy K. Tessema R. A. Budnik L. T. Adam B. (2019). Comparative cyto- and genotoxicity assessment of glyphosate and glyphosate-based herbicides in human peripheral white blood cells. Environ. Res. 179 (Pt B), 108851. 10.1016/j.envres.2019.108851 31678731

[B94] Narimani M. da Silva G. (2020). Thermal decomposition kinetics of glyphosate (GP) and its metabolite aminomethylphosphonic acid (AMPA). Environ. Sci. Process Impacts 22 (1), 152–160. 10.1039/c9em00422j 31778134

[B95] Noori J. S. Dimaki M. Mortensen J. Svendsen W. E. (2018). Detection of glyphosate in drinking water: a fast and direct detection method without sample pretreatment. Sensors (Basel) 18 (9), 2961. 10.3390/s18092961 30189680 PMC6163928

[B96] Pandey A. Dhabade P. Kumarasamy A. (2019). Inflammatory effects of subacute exposure of roundup in rat liver and adipose tissue. Dose Response 17 (2), 1559325819843380. 10.1177/1559325819843380 31205454 PMC6537504

[B97] Pandher U. Kirychuk S. Schneberger D. Thompson B. Aulakh G. Sethi R. S. (2021a). Lung inflammation from repeated exposure to LPS and glyphosate. Cell Tissue Res. 386 (3), 637–648. 10.1007/s00441-021-03531-7 34626244

[B98] Pandher U. Kirychuk S. Schneberger D. Thompson B. Aulakh G. Sethi R. S. (2021b). Pulmonary inflammatory response from co-exposure to LPS and glyphosate. Environ. Toxicol. Pharmacol. 86, 103651. 10.1016/j.etap.2021.103651 33812014

[B99] Pandher U. Kirychuk S. Schneberger D. Thompson B. Aulakh G. Sethi R. S. (2023). Adhesion molecules in lung inflammation from repeated glyphosate exposures. Int. J. Environ. Res. Public Health 20 (8), 5484. 10.3390/ijerph20085484 37107767 PMC10138447

[B100] Panzacchi S. Mandrioli D. Manservisi F. Bua L. Falcioni L. Spinaci M. (2018). The Ramazzini Institute 13-week study on glyphosate-based herbicides at human-equivalent dose in Sprague Dawley rats: study design and first in-life endpoints evaluation. Environ. Health 17 (1), 52. 10.1186/s12940-018-0393-y 29843719 PMC5972408

[B101] Parvez S. Gerona R. R. Proctor C. Friesen M. Ashby J. L. Reiter J. L. (2018). Glyphosate exposure in pregnancy and shortened gestational length: a prospective Indiana birth cohort study. Environ. Health 17 (1), 23. 10.1186/s12940-018-0367-0 29519238 PMC5844093

[B102] Peillex C. Pelletier M. (2020). The impact and toxicity of glyphosate and glyphosate-based herbicides on health and immunity. J. Immunotoxicol. 17 (1), 163–174. 10.1080/1547691X.2020.1804492 32897110

[B103] Perez-Torres I. Zuniga-Munoz A. M. Guarner-Lans V. (2017). Beneficial effects of the amino acid Glycine. Mini Rev. Med. Chem. 17 (1), 15–32. 10.2174/1389557516666160609081602 27292783

[B104] Pham T. H. Derian L. Kervarrec C. Kernanec P. Y. Jegou B. Smagulova F. (2019). Perinatal exposure to glyphosate and a glyphosate-based herbicide affect spermatogenesis in mice. Toxicol. Sci. 169 (1), 260–271. 10.1093/toxsci/kfz039 30785197

[B105] Pierce J. S. Roberts B. Kougias D. G. Comerford C. E. Riordan A. S. Keeton K. A. (2020). Pilot study evaluating inhalation and dermal glyphosate exposure resulting from simulated heavy residential consumer application of Roundup®. Inhal. Toxicol. 32 (8), 354–367. 10.1080/08958378.2020.1814457 32892662

[B106] Pizzino G. Irrera N. Cucinotta M. Pallio G. Mannino F. Arcoraci V. (2017). Oxidative stress: harms and benefits for human health. Oxid. Med. Cell Longev. 2017, 8416763. 10.1155/2017/8416763 28819546 PMC5551541

[B107] Portier C. J. Armstrong B. K. Baguley B. C. Baur X. Belyaev I. Belle R. (2016). Differences in the carcinogenic evaluation of glyphosate between the international agency for research on cancer (IARC) and the European food safety authority (EFSA). J. Epidemiol. Community Health 70 (8), 741–745. 10.1136/jech-2015-207005 26941213 PMC4975799

[B108] Qiu S. Fu H. Zhou R. Yang Z. Bai G. Shi B. (2020). Toxic effects of glyphosate on intestinal morphology, antioxidant capacity and barrier function in weaned piglets. Ecotoxicol. Environ. Saf. 187, 109846. 10.1016/j.ecoenv.2019.109846 31677563

[B109] Rahal A. Kumar A. Singh V. Yadav B. Tiwari R. Chakraborty S. (2014). Oxidative stress, prooxidants, and antioxidants: the interplay. Biomed. Res. Int. 2014, 761264. 10.1155/2014/761264 24587990 PMC3920909

[B110] Rawat D. Bains A. Chawla P. Kaushik R. Yadav R. Kumar A. (2023). Hazardous impacts of glyphosate on human and environment health: occurrence and detection in food. Chemosphere 329, 138676. 10.1016/j.chemosphere.2023.138676 37054847

[B111] Richard S. Moslemi S. Sipahutar H. Benachour N. Seralini G. E. (2005). Differential effects of glyphosate and roundup on human placental cells and aromatase. Environ. Health Perspect. 113 (6), 716–720. 10.1289/ehp.7728 15929894 PMC1257596

[B112] Robinson C P. C. Cavoski A. (2020). Achieving a high level of protection from pesticides in Europe: problems with the current risk assessment procedure and solutions. Eur. J. Risk Regul. 3, 450–480. 10.1017/err.2020.18

[B113] Rueda-Ruzafa L. Cruz F. Roman P. Cardona D. (2019). Gut microbiota and neurological effects of glyphosate. Neurotoxicology 75, 1–8. 10.1016/j.neuro.2019.08.006 31442459

[B114] Samsel A. S. Seneff S. (2016). Glyphosate pathways to modern diseases V: amino acid analogue of glycine in diverse proteins. J. Biol. Phys. Chem. 16 (1), 9–46. 10.4024/03sa16a.jbpc.16.01

[B115] Santovito A. Ruberto S. Gendusa C. Cervella P. (2018). *In vitro* evaluation of genomic damage induced by glyphosate on human lymphocytes. Environ. Sci. Pollut. Res. Int. 25 (34), 34693–34700. 10.1007/s11356-018-3417-9 30324367

[B116] Schwedt I. Schone K. Eckert M. Pizzinato M. Winkler L. Knotkova B. (2023). The low mutational flexibility of the EPSP synthase in Bacillus subtilis is due to a higher demand for shikimate pathway intermediates. Environ. Microbiol. 25 (12), 3604–3622. 10.1111/1462-2920.16518 37822042

[B117] Shin J. Lim N. Roh S. (2020). Severe chemical burns related to dermal exposure to herbicide containing glyphosate and glufosinate with surfactant in Korea. Ann. Occup. Environ. Med. 32, e28. 10.35371/aoem.2020.32.e28 32802344 PMC7406703

[B118] Sidthilaw S. Sapbamrer R. Pothirat C. Wunnapuk K. Khacha-Ananda S. (2022). Effects of exposure to glyphosate on oxidative stress, inflammation, and lung function in maize farmers, Northern Thailand. BMC Public Health 22 (1), 1343. 10.1186/s12889-022-13696-7 35836163 PMC9281059

[B119] Simonetti E. Cartaud G. Quinn R. M. Marotti I. Dinelli G. (2015). An interlaboratory comparative study on the quantitative determination of glyphosate at low levels in wheat flour. J. AOAC Int. 98 (6), 1760–1768. 10.5740/jaoacint.15-024 26651590

[B120] Singh S. Kumar V. Gill J. P. K. Datta S. Singh S. Dhaka V. (2020). Herbicide glyphosate: toxicity and microbial degradation. Int. J. Environ. Res. Public Health 17 (20), 7519. 10.3390/ijerph17207519 33076575 PMC7602795

[B121] Steinborn A. Alder L. Michalski B. Zomer P. Bendig P. Martinez S. A. (2016). Determination of glyphosate levels in breast milk samples from Germany by LC-MS/MS and GC-MS/MS. J. Agric. Food Chem. 64 (6), 1414–1421. 10.1021/acs.jafc.5b05852 26808680

[B122] Tang Q. Tang J. Ren X. Li C. (2020). Glyphosate exposure induces inflammatory responses in the small intestine and alters gut microbial composition in rats. Environ. Pollut. 261, 114129. 10.1016/j.envpol.2020.114129 32045792

[B123] Tarazona J. V. Court-Marques D. Tiramani M. Reich H. Pfeil R. Istace F. (2017). Glyphosate toxicity and carcinogenicity: a review of the scientific basis of the European Union assessment and its differences with IARC. Arch. Toxicol. 91 (8), 2723–2743. 10.1007/s00204-017-1962-5 28374158 PMC5515989

[B124] Tarmure S. Alexescu T. G. Orasan O. Negrean V. Sitar-Taut A. V. Coste S. C. (2020). Influence of pesticides on respiratory pathology - a literature review. Ann. Agric. Environ. Med. 27 (2), 194–200. 10.26444/aaem/121899 32588592

[B125] Tassinari V. Smeriglio A. Stillittano V. Trombetta D. Zilli R. Tassinari R. (2023). Endometriosis treatment: role of natural polyphenols as anti-inflammatory agents. Nutrients 15 (13), 2967. 10.3390/nu15132967 37447296 PMC10343861

[B126] Thongprakaisang S. Thiantanawat A. Rangkadilok N. Suriyo T. Satayavivad J. (2013). Glyphosate induces human breast cancer cells growth via estrogen receptors. Food Chem. Toxicol. 59, 129–136. 10.1016/j.fct.2013.05.057 23756170

[B127] Torretta V. Viotti P. Rada E. C. (2018). Critical review of the effects of glyphosate exposure to the environment and humans through the food supply chain. Sustainability 10, 950. 10.3390/su10040950

[B128] Travlos I. Cheimona N. Bilalis D. (2017). Glyphosate efficacy of different salt formulations and adjuvant additives on various weeds. Agronomy 7 (60), 60. 10.3390/agronomy7030060

[B129] Unlu Endirlik B. Bakir E. Okcesiz A. Guler A. Hamurcu Z. Eken A. (2022). Investigation of the toxicity of a glyphosate-based herbicide in a human liver cell line: assessing the involvement of Nrf2 pathway and protective effects of vitamin E and α-lipoic acid. Environ. Toxicol. Pharmacol. 96, 103999. 10.1016/j.etap.2022.103999 36252731

[B130] Van Bruggen A. H. C. He M. M. Shin K. Mai V. Jeong K. C. Finckh M. R. (2018). Environmental and health effects of the herbicide glyphosate. Sci. Total Environ. 616-617, 255–268. 10.1016/j.scitotenv.2017.10.309 29117584

[B131] Vandenberg L. N. Blumberg B. Antoniou M. N. Benbrook C. M. Carroll L. Colborn T. (2017). Is it time to reassess current safety standards for glyphosate-based herbicides? J. Epidemiol. Community Health 71 (6), 613–618. 10.1136/jech-2016-208463 28320775 PMC5484035

[B132] Vona R. Cappelletti M. Severi C. Matarrese P. (2021). The impact of oxidative stress in human pathology: focus on gastrointestinal disorders. Antioxidants 10, 201. 10.3390/antiox10020201 33573222 PMC7910878

[B133] WHO/FAO (2004). “Pesticide residues in food - 2004: toxicological evaluations,” in Joint meeting of the FAO panel of experts on pesticide Residues in food and the environment and the WHO core assessment group. W.H.O.F.a.A.O.o.t.U. Nations).

[B134] WHO/FAO (2016). Pesticide residues in food 2016.

[B135] Williams G. M. Aardema M. Acquavella J. Berry S. C. Brusick D. Burns M. M. (2016). A review of the carcinogenic potential of glyphosate by four independent expert panels and comparison to the IARC assessment. Crit. Rev. Toxicol. 46 (Suppl. 1), 3–20. 10.1080/10408444.2016.1214677 27677666

[B136] Williams G. M. Kroes R. Munro I. C. (2000). Safety evaluation and risk assessment of the herbicide Roundup and its active ingredient, glyphosate, for humans. Regul. Toxicol. Pharmacol. 31 (2 Pt 1), 117–165. 10.1006/rtph.1999.1371 10854122

[B137] Wozniak E. Reszka E. Jablonska E. Balcerczyk A. Broncel M. Bukowska B. (2020). Glyphosate affects methylation in the promoter regions of selected tumor suppressors as well as expression of major cell cycle and apoptosis drivers in PBMCs (*in vitro* study). Toxicol Vitro 63, 104736. 10.1016/j.tiv.2019.104736 31751608

[B138] Wozniak E. Reszka E. Jablonska E. Michalowicz J. Huras B. Bukowska B. (2021). Glyphosate and AMPA induce alterations in expression of genes involved in chromatin architecture in human peripheral blood mononuclear cells (*in vitro*). Int. J. Mol. Sci. 22 (6), 2966. 10.3390/ijms22062966 33803994 PMC7998550

[B139] Wozniak E. Sicinska P. Michalowicz J. Wozniak K. Reszka E. Huras B. (2018). The mechanism of DNA damage induced by Roundup 360 PLUS, glyphosate and AMPA in human peripheral blood mononuclear cells - genotoxic risk assessement. Food Chem. Toxicol. 120, 510–522. 10.1016/j.fct.2018.07.035 30055318

[B140] Ye M. Beach J. Martin J. W. Senthilselvan A. (2013). Occupational pesticide exposures and respiratory health. Int. J. Environ. Res. Public Health 10 (12), 6442–6471. 10.3390/ijerph10126442 24287863 PMC3881124

[B141] Yoshioka N. Asano M. Kuse A. Mitsuhashi T. Nagasaki Y. Ueno Y. (2011). Rapid determination of glyphosate, glufosinate, bialaphos, and their major metabolites in serum by liquid chromatography-tandem mass spectrometry using hydrophilic interaction chromatography. J. Chromatogr. A 1218 (23), 3675–3680. 10.1016/j.chroma.2011.04.021 21530973

[B142] Zielen S. Trischler J. Schubert R. (2015). Lipopolysaccharide challenge: immunological effects and safety in humans. Expert Rev. Clin. Immunol. 11 (3), 409–418. 10.1586/1744666X.2015.1012158 25655761

[B143] Zobiole L. H. S. Bonini E. A. de Oliveira R. S. Kremer R. J. Ferrarese-Filho O. (2010). Glyphosate affects lignin content and amino acid production in glyphosate-resistant soybean. Acta Physiol. Plant 32, 831–837. 10.1007/s11738-010-0467-0

[B144] Zouaoui K. Dulaurent S. Gaulier J. M. Moesch C. Lachatre G. (2013). Determination of glyphosate and AMPA in blood and urine from humans: about 13 cases of acute intoxication. Forensic Sci. Int. 226 (1-3), e20–e25. 10.1016/j.forsciint.2012.12.010 23291146

